# Voxel Scale Complex Networks of Functional Connectivity in the Rat Brain: Neurochemical State Dependence of Global and Local Topological Properties

**DOI:** 10.1155/2012/615709

**Published:** 2012-07-31

**Authors:** Adam J. Schwarz, Alessandro Gozzi, Alessandro Chessa, Angelo Bifone

**Affiliations:** ^1^Neurosciences Centre of Excellence in Drug Discovery, GlaxoSmithKline Medicines Research Centre, via Fleming 4, 37135 Verona, Italy; ^2^Translational Medicine, Lilly Research Laboratories, Eli Lilly and Company, Indianapolis, IN 46285, USA; ^3^Istituto Italiano di Tecnologia, Center for Nanotechnology Innovation @NEST, Piazza San Silvestro 12, 56127 Pisa, Italy; ^4^Istituto dei Sistemi Complessi, Consiglio Nazionale delle Ricerche e Dipartimento di Fisica, Università “Sapienza”, Piazzale A. Moro 2, 00185 Rome, Italy; ^5^Linkalab, Complex Systems Computational Laboratory, 09129 Cagliari, Italy

## Abstract

Network analysis of functional imaging data reveals emergent features of the brain as a function of its topological properties. However, the brain is not a homogeneous network, and the dependence of functional connectivity parameters on neuroanatomical substrate and parcellation scale is a key issue. Moreover, the extent to which these topological properties depend on underlying neurochemical changes remains unclear. In the present study, we investigated both global statistical properties and the local, voxel-scale distribution of connectivity parameters of the rat brain. Different neurotransmitter systems were stimulated by pharmacological challenge (*d*-amphetamine, fluoxetine, and nicotine) to discriminate between stimulus-specific functional connectivity and more general features of the rat brain architecture. Although global connectivity parameters were similar, mapping of local connectivity parameters at high spatial resolution revealed strong neuroanatomical dependence of functional connectivity in the rat brain, with clear differentiation between the neocortex and older brain regions. Localized foci of high functional connectivity independent of drug challenge were found in the sensorimotor cortices, consistent with the high neuronal connectivity in these regions. Conversely, the topological properties and node roles in subcortical regions varied with neurochemical state and were dependent on the specific dynamics of the different functional processes elicited.

## 1. Introduction

Functional connectivity analyses of neuroimaging data aim to elucidate relationships between signals originating in spatially distinct brain regions [1–7] as an indication of coordinated activity in distributed neural systems, an approach that complements the more established univariate approaches in which the responses in each brain region are analyzed independently. Consistent with this emphasis on interactions between distributed brain structures, neuroimaging data can be represented mathematically as a graph, or network, of nodes and links [8–11]. In this framework, image voxels or parcellated brain regions represent the nodes and a measure of similarity in their responses defines the connections between them [7, 12–17]. 

Recent developments in the theory of complex networks have shown that the topological and statistical properties of networks can reveal fundamental behaviors of the systems that they model. For example, “small-world” topology, characterized by dense local clustering and few-long range connections [18, 19], can support connectivity at multiple spatial-scales while minimizing wiring costs. Another class of networks of particular interest is that of “scale-free” networks, that is, graphs presenting a power-law distribution of the node degree—the number of other nodes to which each node is connected. Scale-free networks appear to be almost ubiquitous in real-world situations, including epidemiology, sociology, and cell biology, thus suggesting that their properties satisfy some general principle of efficiency, robustness and cost-effectiveness [18].

Brain functional connectivity is amenable to complex network analysis, and the interest in the statistical properties of these brain networks is growing rapidly [9–11]. The brain presents several features reminiscent of complex networks, including its ability to support both segregated and distributed information processing at multiple scales, its relative robustness to random neuronal loss due to disease or aging, and its efficiency in terms of low energy and wiring costs [20]. Indeed, functional connectivity networks constructed from human fMRI data under a finger tapping paradigm were shown to exhibit scale-free behavior, with the exponent of the power law robust to the specific choice of threshold [13]. Other authors [14] have reported an exponentially truncated power-law distribution for cortical functional connectivity in human subjects. More generally, “small-world” topology has been demonstrated in both “resting-state” functional [14, 16, 21, 22] and anatomical [23, 24] connectivity in the brain. 

However, while global statistical parameters may be informative of large scale connectivity properties, they do not capture the heterogeneity of the brain. Indeed, different anatomical structures that evolved at different times may be characterized by different local network topology. Many studies thus far have employed volume of interest (VOI)-level parcellations and focused on cortico-cortical connections in humans and primates [14, 25–27], with less attention on the organization of subcortical networks and their connections to cortical structures. Recently, a voxel-scale analysis of human resting state data confirmed scale-free and small-world global properties of the resulting brain networks, and showed nodes of highest degree localized to regions in the cingulate and superior temporal cortices as well as the thalamus [28]. However, the extent to which the afore-mentioned graph-theoretic properties hold in the mammalian brain more generally has not, to date, been resolved. Another key question is the manner in which functional network properties are constrained by underlying anatomical connections and the relationship between their topological characteristics—an issue which has begun to be elucidated in humans [29–33], where perturbed resting functional network parameters in chronic disease states [16, 21, 22] may be closely coupled to disruptions in anatomical structure and connectivity [34]. In turn, the extent to which network properties are modulated by the active engagement of specific brain circuits has begun to receive attention [35]. In humans, there is evidence that global network structure is preserved in the presence of modified connectivity relationships due to performance of functional tasks [36, 37] or acute drug exposure [15]. 

In this paper, we report on a complex-network analysis characterizing voxel-scale topological properties of functional connectivity networks in the rat brain under distinct pharmacological conditions. In contrast to human fMRI studies, in which functional connectivity is typically probed in the brain's resting state or in the context of cognitive tasks, we examine characteristics of complex networks derived from the response of the rat brain to acute pharmacological challenge with three canonical drugs with distinct pharmacological mechanisms (*d*-amphetamine, fluoxetine, and nicotine), thus probing the dependence of functional connectivity network parameters on the engagement of different neurotransmitter systems. This approach, based on intersubject correlations, follows a procedure established in 2-DG autoradiography [38] and PET [39, 40] and validated in pharmacological MRI (phMRI) [6, 41–43]. Recent network-theoretic investigations of anatomical networks based on cortical gray matter thickness derived from MRI data have also employed this approach [23, 34]. Our aim was to work with explicit network representations of the data with nodes defined at the voxel rather than a regional parcellation scale and, in addition to node degree (the number of connections from a given node to others), to examine the node clustering coefficient (a measure of “cliquishness” in node connections). We mapped the anatomical distribution of these node parameters at single-voxel resolution to investigate at high spatial resolution how network connectivity depends on anatomical substrate and pharmacological stimulus, and compare with VOI-level summary statistics. These high-resolution neuroanatomical distributions of complex network parameters in the rat brain reveal foci of high connectivity in the sensorimotor cortex but also drug-dependent features in sub-cortical and prefrontal regions; in particular, a disjunct distribution of nodes of highest degree versus those with highest clustering coefficient.

## 2. Methods

### 2.1. MRI Data Acquisition

All experiments were carried out in accordance with Italian regulations governing animal welfare and protection. Protocols were also reviewed and consented to by a local animal care committee, in accordance with the guidelines of the Principles of Laboratory Animal Care (NIH publication 86–23, revised 1985). MRI data were acquired from male Sprague-Dawley rats using a Bruker Biospec 4.7T scanner with a cylindrical volume coil for RF transmit and a Bruker quadrature “rat brain” surface receive coil. PhMRI data were acquired as RARE time series, sensitized to changes in relative cerebral blood volume (rCBV) by administration of a 2.67 mL/kg intravenous bolus of the blood pool contrast agent Endorem (Guerbet, France). Experiments were performed under 0.8% halothane maintenance anesthesia, neuromuscular blockade and artificial ventilation with blood gas values maintained within physiological range (30 < pCO_2_ < 50; pO_2_ > 100), and peripheral blood pressure within the autoregulatory range associated with halothane anesthesia [44, 45]. The data described in this paper originate from three studies, for which acquisition details were substantially similar and which have been published previously [6, 46]. In the first study, the animals were challenged with either *d*-amphetamine (1 mg/kg i.v., *N* = 17) or vehicle (saline, *N* = 7), respectively [6, 42]. In the second, animals were challenged with fluoxetine (10 mg/kg i.p., *N* = 7) [6]. In the third, animals were challenged with nicotine (1 mg/kg i.v., *N* = 9) [46]. In total, complex networks were constructed as detailed below from four subject cohorts: the *d*-amphetamine and vehicle groups in the first study as well as from the fluoxetine and nicotine groups.

### 2.2. Analysis Details

#### 2.2.1. MR Image Preprocessing

Anatomical and time series data were converted to Analyze (AVW 7.5) format and signal intensity changes in each time series were transformed into fractional rCBV on a voxel-wise basis, using a constrained exponential model of the gradual elimination of contrast agent from the blood pool to provide a robust prediction of postinjection background signal and remove the worst effects of this systematic trend in the resulting rCBV data [47]. Data for each subject were then spatially normalized to a stereotaxic rat brain template [48] by computing a nine degree-of-freedom affine transform for the anatomical image and applying the resulting transformation matrix to the accompanying rCBV time series (FSL/FLIRT v.5.2). Finally, the rCBV data were multiplied by a brain parenchyma mask to remove extra-cranial and CSF contributions.

#### 2.2.2. Time Series Analysis

Image-based time series analysis of the response in individual subjects was carried out in a general linear model framework in order to calculate 3D maps of the post-injection response amplitude in each subject. The images were spatially smoothed with a Gaussian kernel of FWHM = 0.6 mm, corresponding to ~2× the in-plane voxel dimension. All image processing was performed with the voxel dimensions scaled up in the image headers by a factor of 10, in order to ensure compatibility with any explicit length scales that may be encoded in algorithms designed for use with human data. However, explicit voxel dimensions are quoted at the original scale. The design matrix for each study comprised a signal model function identified by study-level Wavelet Cluster Analysis (WCA), the temporal derivative of this regressor and a linear ramp [49, 50]. This allows a good model fit to signals whose temporal response profile can vary slightly across subjects and brain regions. 

The coefficients of the signal model function thus provided a map of the post-injection response amplitude for each subject. The response maps for the subjects in each study were then stacked together so that each voxel had an associated response vector. The inter-subject correlations analyzed here leverage the differential anatomical profiles of phMRI response between subjects [6, 42].

### 2.3. Creation of Network Representations

The response maps, calculated at the template dimensions, were rebinned in-plane by a factor of two. This was performed so that subsequent adjacency matrices remained within the memory limits of the IDL software used for much of the processing and also to recover voxel volumes closer to the actual acquisition resolution, since as part of the spatial normalization process the time series' were interpolated to the resolution of the standard space template [48]. The rebinned response maps thus had 0.12 mm^3^ voxels, close to the acquisition resolution size of 0.09 mm^3^. A binary brain mask, covering only slices for which complete data were present for all subjects in all studies, was used to define brain parenchyma voxels for further analysis. This resulted in networks of *N* = 8130 nodes (voxels).

A fully weighted, complete network was created for each study by considering each voxel as a node and defining the strength of the edge between each pair of voxels based on the linear correlation between the response vectors associated with each. Specifically, the weight of each edge *w*_*ij*_ was defined as the absolute value of the Pearson correlation coefficient *r*_*ij*_ between the inter-subject response amplitudes in each voxel, converted to lie under an approximately normal distribution by applying Fisher's *r*-to-*z* transformation:

(1) $$\begin{array}{c} w_{ij}=\left|z_{ij}\right|,\\ z_{ij}=\frac{1}{2}\log\left(\frac{1+r_{ij}}{1-r_{ij}}\right), \end{array}$$

where *i*, *j* ∈ {1,…, *N*_nodes_} specify the pair of nodes connected by each edge. Note that these networks are undirected—each edge simple conveys the strength of a connection without regard to a causal direction. Each of the four weighted networks was then converted into a binary one by retaining only the edges with the highest weights (i.e., representing the strongest connections). This step was performed in order to make networks of this size tractable for further analysis; specifically, calculation of nodewise network parameters is substantially faster for sparse binary networks. Although extension of complex network theory to weighted networks is of considerable current interest, properties of binary networks are well established and previous fMRI network studies have also employed a binarization step. We applied a threshold *z*_thresh_ to the link weights, determined as that which retained the strongest 2% of the *N*_nodes_ × (*N*_nodes_ − 1)/2 edges in the fully weighted network; that is, we worked with equi-sparse networks, ensuring a consistent number of network edges across data sets to emphasize differences in the relative connection topology rather than overall edge density *per se*. This value was empirically determined as one that allows a diversity of node connectivities, whilst retaining a connected network, and is consistent with the thresholding scheme used in our previous seed region and community structure analyses [43]. The network features and in particular the anatomical profiles of the nodewise connectivity parameters were robust across a range of binarization thresholds (see Supplementary Data available online at doi:10.1155/2012/615709). The threshold values *z*_thresh_ are summarized for each of the four networks analyzed in Table 1. 

The resulting binary networks can be represented mathematically by an adjacency matrix *A*, whose elements *a*_*ij*_ describe the connectivity:

(2) $$\begin{array}{c} a_{ij}=\{\begin{array}{cc} 1, & \text{if}\;\;\text{nodes}\;\;i\;\;\text{and}\;\;j\;\;\text{are}\;\;\text{connected}\\ 0, & \text{otherwise}. \end{array} \end{array}$$

### 2.4. Nodewise Network Parameters

Based on the topology defined by the adjacency matrix (2), a number of network parameters can be derived that convey information about the network. Here, we investigate the node degree *k* and the clustering coefficient *c*, as follows.

The degree *k*_*i*_ of any node *i* is simply the number of nodes to which it is connected, that is, the number of edges incident upon it:

(3) $$\begin{array}{c} k_{i}=\sum_{j=1}^{N_{\text{nodes}}}a_{ij}. \end{array}$$

The clustering coefficient *c*_*i*_ is defined as the fraction of total possible edges *N*_edges_(*G*_*i*_) in the sub-network *G*_*i*_, defined by all nodes directly connected to node *i*, that are actually present:

(4) $$\begin{array}{c} c_{i}=\frac{2N_{\text{edges}}\left(G_{i}\right)}{k_{i}\left(k_{i}-1\right)}. \end{array}$$

In other words, this parameter reflects how many pairs of nodes connected to a given node are also connected to each other.

Global, whole-network histograms for the parameters *k* and *c* were generated for each network. These distributions capture global statistical properties of the network and reflect its basic principles of organization. The form of the histogram of node degree *k* is of particular interest and in networks derived from human functional imaging data has been observed to show power law behavior (a straight line when plotted on logarithmic scales) up to a high-degree cutoff [13, 14]. The power law behavior of the networks in the present study was quantified by fitting an equation of the form *y* = *k* ^−*γ*^ to the linear portion of the histogram, where *y* is the frequency (number of nodes in the bin) and *k* is the mean value of each bin in the histogram. 

Each network was further characterized by calculating the following global summary parameters:

-  The power law decay constant from the degree distribution histogram;
-  
*K*, the average degree *k*_*i*_ over all nodes in the network;
-  
*C*, the average clustering coefficient *c*_*i*_ over all nodes in the network.

The presence of long-distance links in random networks results in small values of *L*, the average shortest path between all node pairs, compared, for example, to regular lattices with only next-neighbor links, with *L* scaling as the logarithm of *N* (the total number of nodes). Watts and Strogatz [18] identified a particular class of networks, dubbed “small-world” networks, with comparable values and scaling properties of *L* (*L* ≈ *L*_random_) in the presence of a high degree of local clustering *C* ≫ *C*_random_ (where *L*_random_ and *C*_random_ are the values from equivalent random networks with the same *N*_nodes_ and link density). We thus also report two indices that explicitly compare these properties in the phMRI networks with those in appropriate comparator networks, namely:

- *γ* = (*C*/*C*_random_), a measure of local clustering;
- *σ*_SW_ = (*C*/*C*_random_)/(*L*/*L*_random_), often referred to as the “small world index” [19, 24].

For each phMRI network, we used 10 randomly rewired versions of the network as a comparator null model [51]. All network parameters were calculated using the brain connectivity toolbox in Matlab [52].

In addition, since each node corresponds to a position in the image volume, their anatomical locations in the brain were used to generate voxel-wise maps and profiles by anatomical structure of each of the above parameters. In this way, the dependence of the above parameters on brain region was evaluated for networks associated with each drug.

For the anatomical structure profiling, volumes of interest (VOIs) corresponding to specific brain structures were selected to enable a formal statistical comparison of differences in network parameters suggested by examination of the parameter maps. VOIs were defined bilaterally using a 3D reconstruction of a rat brain atlas coregistered with the anatomical MRI template [48]. The VOIs selected were: caudate putamen, cingulate cortex, insular cortex, medial prefrontal cortex, parietal association cortex, visual cortex, anterodorsal hippocampus, subiculum, ventral hippocampus, primary motor cortex, whisker barrel field of the primary somatosensory cortex, forelimb field of the primary somatosensory cortex, dorsolateral thalamus, midline dorsal thalamus and ventromedial thalamus.

## 3. Results

### 3.1. Global Network Properties

We first examined the global characteristics of the rat brain networks. A summary of the global parameters is provided in Table 1. Values of the parameters *γ* and *σ*_SW_ were substantially greater than unity and consistent with “small-world” behavior, whereby any one node is connected to any other node in the network by a far fewer number of edges than in a random network with the same overall number of nodes and edges. 

**Figure 1:**
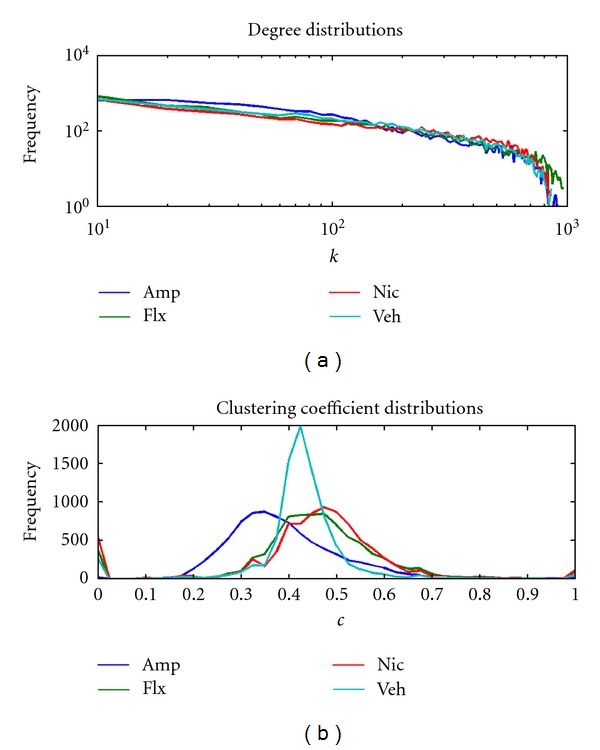
Global parameter histograms. (a) Histograms of the node degree (*k*) reveal a power law dependence of frequency on *k* (evident as a linear relationship in this log-log display) up to a high-frequency cutoff for all networks. The slope of the linear portion of the histograms is similar in each network as indicated by the lines of linear fit (shown here offset below the data for visual clarity) and the values of *γ* in Table 1. Note that the histogram for the vehicle network reaches zero at a lower value than the three drug challenge networks, indicating fewer nodes with very high degree. (b) Histograms of the clustering coefficient (*c*) for each network reveal an increased spread of values for each of the active drug networks relative to the vehicle network.

The degree histograms for all four of the phMRI networks exhibited power law (count ~*k* ^−*γ*^) behavior, characterized by a near linear dependence of frequency on *k* when displayed on a log-log plot, up to a high-*k* cut-off of *k*~ 800–900 (Figure 1(a)). The presence of a cut-off in the distribution of *k*reflects the finite size of the networks. The decay parameters *γ* were similar in each case, ranging from −0.67 for the nicotine network to −1.03 for the amphetamine network (Table 1). 

We also examined histograms of the clustering coefficient *c* for each network (Figure 1(b)). While some differences in the median values across networks were observed (Table 1), we also found strong drug-dependent changes in the *distribution* of *c* values; in particular, each of the three active-drug networks evidenced a profile distinct from the vehicle network and indicated drug-dependent increases and decreases in *c*. While all three active drug networks showed a broader spread of *c* values compared with vehicle, the amphetamine network contained more nodes with lower values of *c*, whereas the fluoxetine and nicotine networks contained more nodes with higher values of *c*.

### 3.2. Anatomical Dependence of the Local Connectivity Parameter *k*

**Figure 2:**
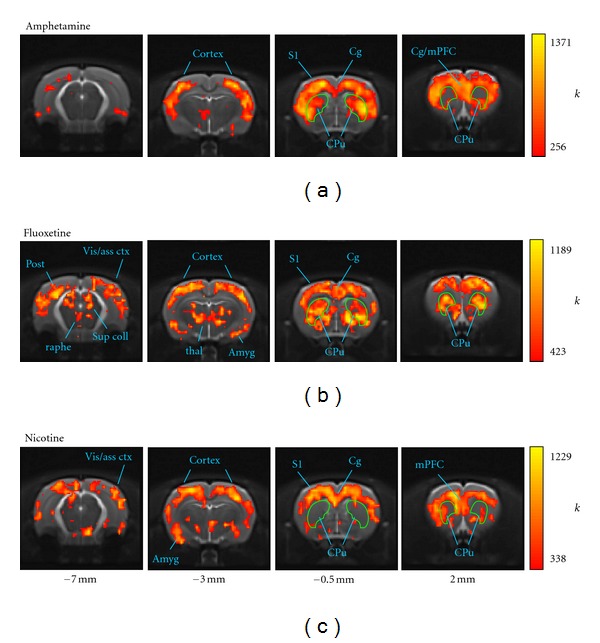
Anatomical distributions of node degree *k*. (a) amphetamine, (b) fluoxetine, and (c) nicotine phMRI networks. In order to highlight the neuroanatomical correspondence of the most highly connected nodes, the overlay shows the upper quartile (75%–100%) of the *k* distribution for each network (the color scale maxima are compressed slightly to optimize the dynamic range.)

We next examined the anatomical dependence of node degree and clustering coefficient, both by mapping these parameters nodewise back onto the anatomical brain template and by statistical comparison with the vehicle network in selected brain structures of interest. The maps of the node degree *k* revealed a strong dependence of connectivity on brain region in all of the phMRI networks (Figure 2).

In the amphetamine network, voxels with the highest values of *k* were localized in particular to frontal and prefrontal cortical regions, including the orbitofrontal, medial prefrontal, cingulate, insular, motor, and somatosensory cortex (Figure 2(a)). Sub-cortical regions containing highly-connected voxels included parts of the striatum (caudate putamen and accumbens), structures in the ventromedial thalamus and medial hypothalamus, with small foci also in the regions of the ventral subiculum and lateral entorhinal cortex. 

In the fluoxetine network (Figure 2(b)), voxels in cortical regions were also characterized by high *k*, but there were substantially more highly-connected sub-cortical nodes evidenced by high-connectivity nodes in the caudate putamen, amygdala and more extensively in the thalamus. Midbrain regions, including parts of the superior colliculi, periaqueductal grey, and medioventral nodes consistent with the raphe nuclei were also highly connected in the fluoxetine network.

The nicotine network also evidenced high connectivity in prefrontal and frontal cortices, parietal association cortex, with focal high-*k* subcortical foci within the thalamus, hypothalamus and amygdala (Figure 2(c)). 

In contrast, the anatomical distribution of *k* in the vehicle network was scattered with a noisier and overall less symmetric appearance than for the networks derived from the three psychoactive drugs (see Supplementary Data)—only slight anatomical dependence was evident with regions of relatively higher connectivity including the medial prefrontal cortex, and nodes within the thalamus and ventral hippocampus/entorhinal cortical regions. 

### 3.3. Anatomical Dependence of the Clustering Coefficient *c*

**Figure 3:**
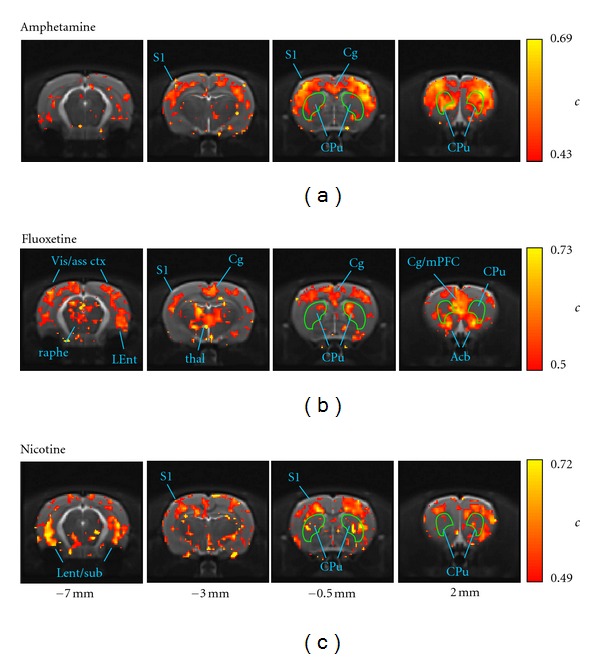
Anatomical distributions of node clustering coefficient *c*. (a) amphetamine, (b) fluoxetine, and (c) nicotine phMRI networks. In order to highlight the neuroanatomical correspondence of the most “cliquish” nodes, the overlay shows the upper quartile (75%–100%) of the *c* distribution for each network (the color scale maxima are compressed slightly to optimize the dynamic range.)

The anatomical distributions of the cluster coefficient *c* are shown in Figure 3. Again, similarities and differences in the anatomical features across networks are evident. 

In the amphetamine network (Figure 3(a)), the regions of high *c* showed some commonality with those of high *k* (cf. Figure 2(a))—in particular in frontal (somatosensory, motor) and prefrontal cortices. However, high-*k* foci in the ventromedial thalamus and hypothalamus were not evident in the *c* map. Moreover, in the caudate putamen, high-*c* nodes were found more rostrally than high-*k* nodes.

In the fluoxetine network (Figure 3(b)), the regions of high-*c* in the frontal slices were more medial and preferentially localized to the mPFC and accumbens, in contrast to the *k* map where the high-*k* voxels were distributed across the motor cortex and more widely in the caudate putamen. Compared with the amphetamine network, more high-*c* nodes were localized in more caudal regions, including the midbrain areas identified above.

In the nicotine network, regions of high-*c* appear less well-defined than those of high-*k*, but high-*c* regions in the more dorsal sensorimotor cortices and ventral hippocampus/entorhinal cortex are evident (Figure 3(c)). Interestingly, the medial and prefrontal cortices, identified in Figure 2 as high-*k* regions, are not regions of high *c* in this network.

The vehicle network showed few anatomically meaningful regions of high *c*, with the exception of the medial prefrontal cortex and entorhinal cortex (Supplemental Data).

### 3.4. Differences between Drug and Vehicle Networks by Anatomical Region

**Figure 4:**
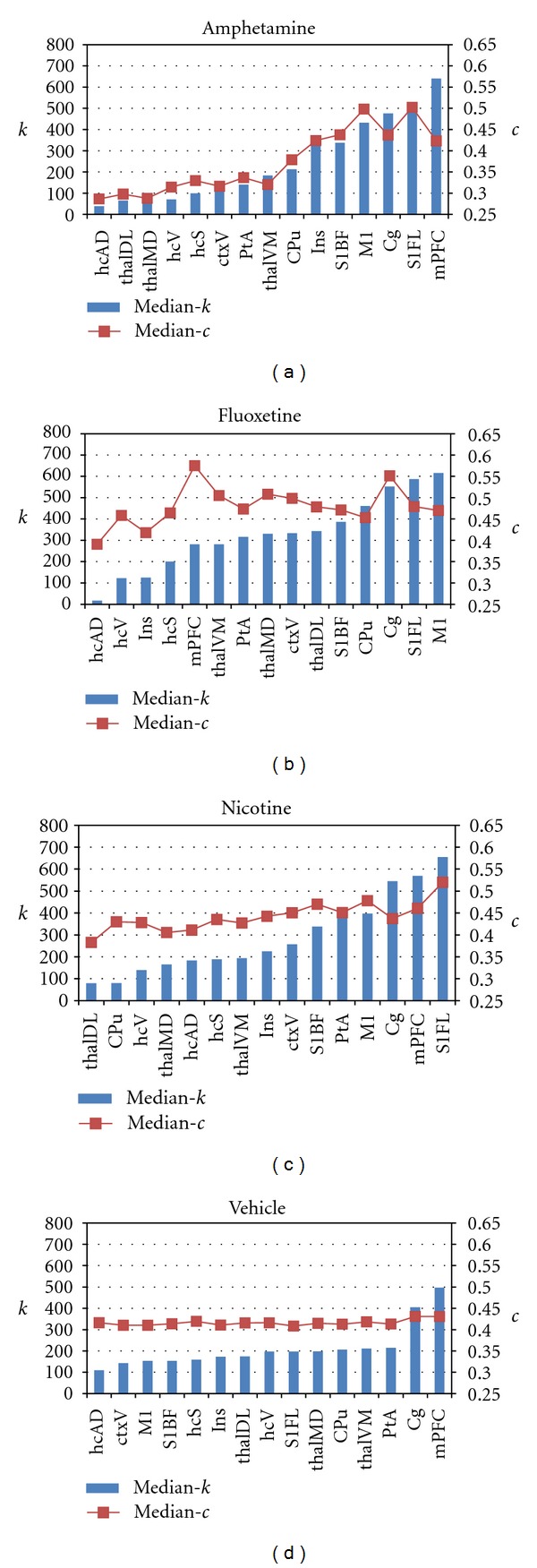
VOI profiles of network parameters. Selected VOIs rank ordered by degree (median *k* value in each VOI) for each network. Amphetamine, fluoxetine, and nicotine networks (a–c) show both increases and decreases in *k* and *c* relative to the mainly flat VOI profile of the vehicle network (d). Rank ordering of both parameters is similar for amphetamine and nicotine, but less closely coupled for the fluoxetine network (see Table 2 for VOI abbreviation definitions.)

We also examined the anatomical profiles of *k* and *c* across selected VOIs representing brain structures of interest.  Figure 4 illustrates the differences in connectivity structure between the active drug networks and the vehicle network. Consistent with the nodewise maps reported above, the latter was characterized by relatively flat profiles of both parameters with the exception of the mPFC and cingulate cortex (Figure 4(d)). In contrast, the other three networks show clear shifts, as a function of both brain region and drug, in the values of both *k* and *c* (Figures 4(a), 4(b), and 4(c)). While some brain regions exhibited an increase in connectivity in the active pharmacological state, in others the values were decreased relative to vehicle.

We further examined these differences in anatomical profile of *k* and *c* at the VOI level by statistical comparisons between the node parameter values within each brain structure for each psychoactive drug network compared to vehicle (Mann-Whitney tests). Results for the node degree *k* are summarized in Table 2. The fluoxetine network had the highest connectivity in thalamic regions, and a differential distribution of *k* across different hippocampal regions. In contrast, the amphetamine network exhibited very low connectivity within the thalamic and hippocampal regions and a differential distribution across cortical regions. Profiles of *c* by VOI for each of the amphetamine, fluoxetine, and nicotine networks also confirmed the differential anatomical dependence indicated by the parameter maps (Table 3). Common differences from vehicle across all three psychoactive drug networks were increased *k* in cingulate, motor and somatosensory cortices, and increased *c* in motor and somatosensory cortices. In other brain regions—in particular subcortically—the anatomical profiles were drug-dependent.

### 3.5. Neuroanatomical Differences between High-*k* and High-*c* Foci

**Figure 5:**
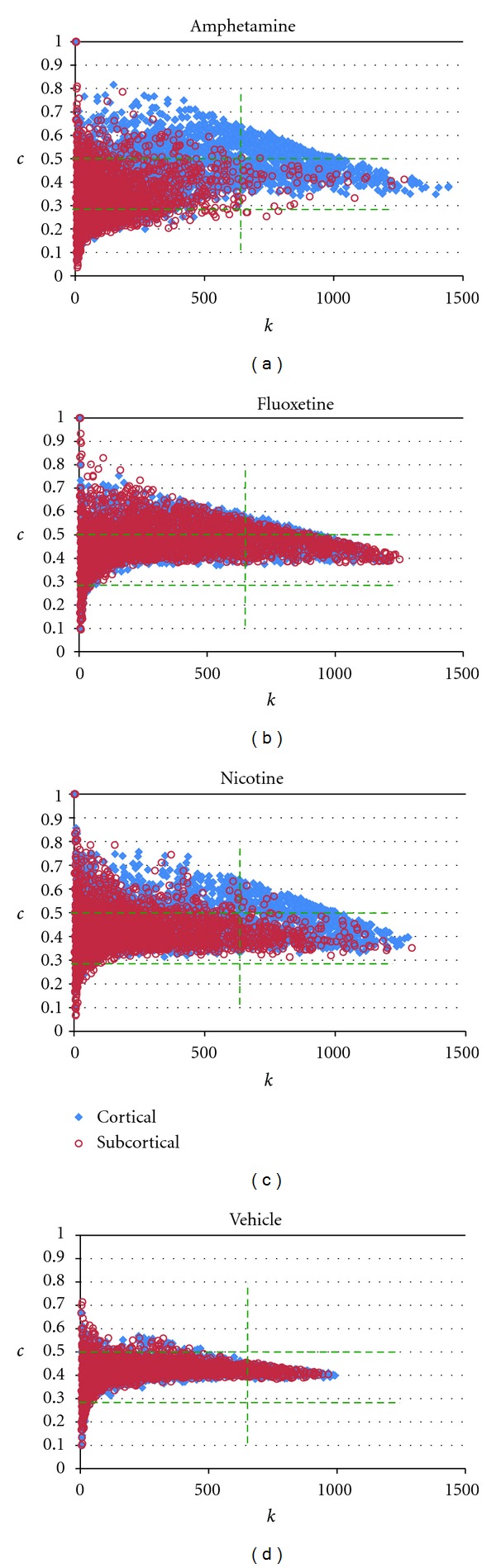
Scatter plots of *c* versus *k* for all nodes, at the voxel scale. Overall, low degree (*k*) nodes were associated with a spread of clustering (*c*) behaviors, whereas the nodes of highest degree had clustering coefficients toward the middle of the range. Compared with vehicle (d), the active-drug networks (a–c) had a greater range in both *k* and *c*. Amphetamine and nicotine showed a differential shift in the *k*-*c* distributions between cortical and subcortical nodes, whereas for fluoxetine nodes in both brain subdivisions were shifted to higher values. The green lines indicate cutoff values used to identify nodes whose *k* and/or *c* characteristics were outside the range associated with the vehicle state (see text).

At the scale of individual nodes (voxels), graphs of *c* versus *k* (Figure 5) revealed that these two parameters were not related by a simple monotonic dependence. For all four networks, there was a far greater range of values of *c* at lower values of *k*. The vehicle network (Figure 5(d)) was characterized by a spread in *c* values between ~0.1 and 0.7 at low *k*, with the range of *c* converging to a value ~0.4 as *k* approached a maximum value ~1000. In contrast, for each of the three active drug networks the distribution extended over a greater range in both *k* and *c*. Dividing the brain into cortical and sub-cortical nodes revealed that in the amphetamine and nicotine networks, cortical nodes were shifted toward higher *k* and *c*, whereas in the amphetamine network in particular the sub-cortical nodes were shifted toward lower values of both *k* and *c* (Figures 5(a) and 5(c)). In contrast, both cortical and sub-cortical nodes in the fluoxetine network were characterized by a greater extent toward higher values of *k* and *c*, with little decrease in either parameter relative to vehicle (Figure 5(b)). For all networks, nodes of highest degree were not those with highest cliquishness, as represented by the clustering coefficient. 

**Figure 6:**
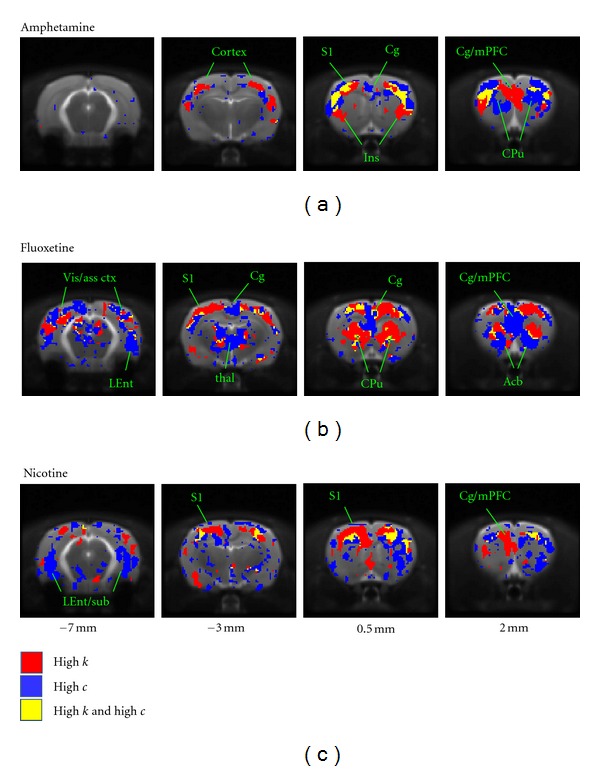
Maps of high-*k* and high-*c* foci for each drug network. These maps were created using cutoff values of *k* and *c* based on the vehicle parameter distributions (see Figure 5). The maps illustrate the anatomical disjunction between nodes of highest clustering, compared with those of highest degree. The neuroanatomical dependence of these is a function of the applied pharmacological challenge for prefrontal and subcortical regions, but similar across networks in the sensorimotor cortex.

Building on the above observations of differences between active drug and vehicle networks at the VOI scale, we anatomically mapped nodes of altered connectivity at the finer neuroanatomical scale offered by the individual voxel nodes. Using the vehicle network as representing a baseline physiological state, we determined common cutoff values of *k* and *c* for the three active drug networks. The 95th percentile of the vehicle *k* distribution and the 2.5th and 97.5th percentiles of the vehicle *c* distribution yielded cutoff values of *k* > 657, *c* < 0.28, and *c* > 0.50, shown as dashed lines in Figure 5. The con/disjunction maps depicted in Figure 6 highlight the voxels in which the nodes of highest *k* and those of highest *c* are localized for each network and indicate the presence of heterogeneity on a finer spatial scale than VOI-scale parcellation schemes. 

Interestingly, nodes with highest values of *c* were, in general, differentially localized from those with the highest values of *k*. For all three drug networks, high-*k* and high-*c* nodes were identified within the sensorimotor cortex, with the greatest amount of overlap for the amphetamine network. Clear boundaries between high-*k* and high-*c* nodes separated the sensorimotor and prefrontal/cingulate cortices in all networks (Figure 6). In the amphetamine network, the mPFC was dominated by high-*k* nodes, whereas a portion of the cingulate cortex more caudally was a high-*c* focus. In the fluoxetine network, both mPFC and cingulate contained high-*c* foci, whereas in the nicotine network the mPFC and cingulate were high-*k* regions. The fluoxetine network showed an interesting differential distribution between the dorsal (CPu) and ventral (nucleus accumbens) striatum, with the former dominated by nodes of high degree whereas the latter featured nodes of high clustering coefficient. More caudally, the fluoxetine and nicotine networks had high-*c* foci in entorhinal cortex/ventral hippocampal regions along with midbrain structures. 

The low-*c* nodes were localized to sub-cortical and midbrain regions in the amphetamine network, consistent with preferential localization of the high-*k* and high-*c* regions to frontocortical brain regions (see Supplementary Data). Low-*c* nodes for the fluoxetine and nicotine networks were few and localized primarily to the brain edge (see Supplementary Data). 

## 4. Discussion

There is increasing interest in using functional imaging techniques to probe connectivity properties of the brain. In this context, it can be intuitive to think of the imaging data—and by extension the brain—as a network, comprising a set of nodes with functional connections defined by links between them. Beyond the conceptual tractability of such a representation, this approach also enables concepts in network theory to be leveraged. The present study extends the application of complex network theory in functional imaging of the rat brain by examining global as well as voxel scale, nodewise, network parameters for data sets characterizing the response to three canonical psychoactive drugs differing in both clinical/behavioral profile and mechanism of action.

Whole-network histograms of connectivity parameters can reveal statistical properties of the network that have deep repercussions for its behavior. Moreover, changes in connectivity properties may also reflect disease states—for example, the global network-mean clustering coefficient and small-world index have been shown to be reduced in Alzheimer's Disease relative to normal aging [16, 22]. For all four of the phMRI networks considered here, whole-brain histograms of *k* demonstrated the characteristic scale-free signature up to a high-*k* cutoff as previously reported for human brain motor task [13] and resting state [14] data. The form of these distributions was robust to the binarization threshold and independent of the challenge drug. A distribution of this type is qualitatively different from that found with random networks, where the degree distribution is strongly unimodal [53]. Heavy-tailed, scale-free degree distributions reflect the presence of a significant number of highly connected nodes, or “hubs,” a characteristic that results in short average distance between any two nodes of the network, and in robustness to random failure of nodes [53]. This is consistent with our observation in the present study of highly-connected brain regions in both cortical and sub-cortical structures in all three active drug networks. Scale-free characteristics have been found in several network analyses of functional imaging data in humans [13, 14]. However, structural studies, based, for example, on diffusion MRI tractography [26], tend to find more exponential distributions (linear on a log-linear scale), consistent with the relatively uniform distribution of neuronal density in grey matter [54]. Hence, it may be argued that the presence of highly connected hubs reflects functional organization of the brain, rather than the structure of the neuronal substrate [10].

In the present study, the anatomical distributions of two key nodewise connectivity parameters—the node degree *k* and the clustering coefficient *c*—revealed bilaterally symmetric patterns whose features correlated well with known anatomical subdivisions of the brain—including, for example, sensorimotor, cingulate, and prefrontal cortices (see Figures 2, 3, and 6). These patterns revealed a common increase in connectivity in sensorimotor cortical regions but a dependence on the challenge drug elsewhere in the brain. A statistical analysis of nodes grouped into anatomical VOIs demonstrated significant differences from vehicle in median *k* and *c* within large-scale brain structures. Interestingly, both increases and decreases in connectivity relative to vehicle were observed (Figures 4 and 5). This is consistent with a preferential functional engagement of certain interregional connections and a suppression of others in the pharmacologically active states. The presence of common features of the anatomical distribution in the sensorimotor cortex for all node-parameters investigated (high *k* and high *c*) suggests that these reflect a general functional or structural organization of the rat brain, consistent with the high local connectivity of cortical grey matter. 

Regions of high *k* reflect voxels that are functionally connected to many others. Studies in which clusters of functional connections in these data were elucidated [6, 42, 43, 55–57] indicate that the voxels in the sensorimotor cortices are likely to be preferentially connected to each other, consistent with the widespread distribution of high-*k* nodes in the cortex for all three drug networks. However, foci of high node degree were also observed as a function of challenge drug in subcortical structures including the thalamus and striatum, and midbrain regions including the raphe nucleus. The clustering coefficient *c* reflects the extent to which the nodes connected to a given node are interconnected within themselves and can be interpreted as an index of local connectivity (where “local” is defined by connections and does not necessarily coincide with anatomical locality) and also representative of local information transfer efficiency [15, 58]. For the *d*-amphetamine network, the anatomical profiles of *k* and *c* both had a strong cortical localization, consistent with a strong cortical subnetwork [42]. In contrast, for fluoxetine regions of high *c* were more localized to prefrontal/cingulate cortices and sub-cortical structures such as the thalamus. This is consistent with the observation of a large sub-network involving these structures by both seed region analysis [6] and network partitioning approaches [43, 55]. The nicotine network also showed widespread elevated node degree in cortical regions, but less clear anatomical structure in the maps of the clustering coefficient. Nevertheless, the VOI analysis demonstrated a different anatomical profile of both *k* and *c* in the response to nicotine, in comparison with the other drug networks. Overall, the high-*k* and high-*c* nodes shown in Figure 6 lie within the communities of “core” nodes identified in a network partitioning analysis of the same data [55].

In networks such as those considered here, with connections based on the response to the injection of a pharmacological agent, comparison with a vehicle network is valuable. 

Explicit comparison with a vehicle group is standard practice in more traditional group analysis approaches in order to differentiate the effects of the pharmaceutical compound *per se* from those due to the solvent in which it is dissolved. Ideally a benign vehicle, such as physiological saline in the group analyzed here, is used and expected to elicit minimal response. Nevertheless, in addition to capturing physiological “baseline” variation in the time courses, vehicle injection may itself give rise to weak effects. In the present context, this allows network structure arising from the intravenous injection of the vehicle to be characterized and used as a baseline for determining effects due to the compounds of interest. In the vehicle group analyzed herein, an intravenous injection volume of 1 mL/kg was used, along with a 0.3 mL/kg flush, yielding a total injection volume of 1.3 ml/kg, injected over one minute. For a 300 g rat, assuming a blood volume of 18.77 mL, this equates to ~7% of the total blood volume. When using blood pool contrast agents as in the CBV method employed in the present study, this results in a slight dilution of the agent which can manifest as a small central signal change post-injection. The injection may also give rise to an autonomic response whose response in particular brain regions may manifest as correlated signal changes, albeit of small amplitude. The network analysis showed weaker anatomical features of connectivity parameters in the vehicle network than in the other three, with elevated *k* and *c* observed in prefrontal cortical regions. Indeed, the global degree distribution of the vehicle network had a scale-free structure very similar to the other phMRI networks, consistent with findings of preserved global topological structure in the presence of cognitively and pharmacologically induced perturbations in (local) functional connectivity in human brain imaging studies [15, 36, 37]. 

Nevertheless, the connectivity characteristics in the active drug networks were qualitatively different from the vehicle network, as evident from Figures 4–6. Indeed, using the vehicle network as a quantitative reference, regions of both high-*k* and high-*c* were clearly identified for all three active drug networks (Figures 5 and 6). These indicated the presence of foci in frontal cortices as a common feature across drugs. Moreover, by considering nodes at the scale of individual voxels, we were able to resolve an anatomical differentiation between the nodes with highest *k* and those with highest *c* at a finer spatial scale than that of typical VOI parcellations. While the use of more liberal cutoff values in Figure 6 would result in increased overlap between high-*k* and high-*c* nodes, the selected values convey that nodes with highest values of *k* do not, in general, coincide with nodes of highest *c*—a finding implied also in the scatter plots depicted in Figure 5. Furthermore, the maps in Figure 6 also illustrate how the highest-*k* and highest-*c* nodes often occupy anatomically adjacent brain structures, particularly in frontal cortical, prefrontal cortical and striatal regions. Sub-cortical foci were also identified, in particular in the fluoxetine network. In contrast, few sub-cortical high-*k* or high-*c* nodes were identified for the amphetamine network at the thresholds used here, despite the presence of a sub-network from the VTA projecting forward to the ventral forebrain and the mPFC [6, 42, 55]; this may reflect the smaller size of this module, or potentially a “sequential” nature of connections along this highly localized pathway (see, e.g., Figure 3 in [6]), which would also tend to produce lower *k* and *c* values.

As in previous studies examining network characteristics of functional imaging networks, we reduced each complete, weighted network (in which all possible links exist and have a variable weight dependent on the correlation in response between its two nodes) to a binary one. The primary reason for this was computational tractability for the relatively large (ca. 10^4^ nodes) networks that resulted from retaining the spatial scale of functional image voxels. For the main results presented here, we thresholded each network so as to retain the strongest 2% of the edges in the binarized version. The resulting network topology represents a middle ground between two undesirable extremes: as more edges are retained, node connections become increasingly dense and topological distinction is lost; alternatively, as fewer edges are retained, the network becomes disconnected and topological information becomes increasingly suppressed. In fact, the global network properties and the relative anatomical distribution of the network parameters *k* and *c* are robust to the precise value of the binarization threshold over a range of thresholds in this “intermediate” regime of interest (see Supplementary Data). Furthermore, the 2% networks at the voxel scale satisfy the *K* > ln⁡(*N*_nodes_) criteria for estimable topological properties in random comparator networks of the same size [15].

In human studies, brain functional connectivity is typically derived from fMRI series by calculating correlations in the time domain. Recent progress has also been made toward establishing robust and reproducible temporal correlation patterns in rodent fMRI [59–64]. Based on the nature of the present phMRI data, acquired with a lower temporal resolution than required to resolve temporal correlations, we constructed and characterized networks derived from inter-subject correlations in the response amplitude following drug administration, following a procedure established in 2DG autoradiography [38] and PET [39, 40]. The correlated responses used to determine the links can be interpreted as reflecting a functional coupling in response to the pharmacological challenge in each case [38]. It should be noted that the concept of functional connectivity was first introduced in this context, based on inter-subject correlation analysis and prior to the invention of fMRI. Moreover, this approach has been employed recently to elucidate anatomical networks of grey matter volume from structural MRI data [23, 34]. The use of cross-subject correlations to derive functional connectivity from phMRI data has been recently demonstrated [6], shown to delineate functional connectivity along different neurotransmitter systems when they were selectively stimulated pharmacologically, and further validated in subsequent work [42, 55].

The images in this study were smoothed before conversion into the network representation, introducing a local correlation between responses in neighboring voxels. A key reason for smoothing is to compensate in part for residual differences in image alignment between different subjects when performing group-level, voxel-wise operations. In the present data, the networks are derived from inter-subject correlations and so spatial normalization of the image data to a common space is critical. To assess the effect of smoothing, we also performed the analyses on networks derived from unsmoothed image data. Smoothing did not greatly affect global characteristics nor anatomical distribution of parameters; a fine anatomical resolution was maintained (Figures 2, 3, and 6). The results in terms of global and anatomical parameter distributions were highly consistent with those obtained from the smoothed image networks—the most noticeable difference was that the parameter maps appeared noisier and less easy to interpret visually in the unsmoothed case. Thus, to depict the neuroanatomical dependence of the network parameters with maximum clarity, we presented data based on the smoothed images.

An important feature of networks generated from functional imaging data is that each node represents a brain region and has a well-defined neuroanatomical location. Here, we investigated the dependence of node degree and clustering coefficient in response to different pharmacological stimulation in the rat. Global degree histograms indicated similar global, scale-free structure in all networks. However, nodewise maps at the scale of functional image voxels and VOI-level comparisons of these parameters revealed drug- and brain region-dependent modulation of connectivity parameters relative to the physiological baseline state. Increases in both node degree and clustering coefficient in frontal cortices were observed for all active-drug networks, revealing foci of high connectivity independent of the pharmacological challenge. Sub-cortical and pre-frontal features were stimulus-dependent, and showed a disjunct distribution of nodes of highest degree versus those with highest clustering coefficient. These findings suggests that the cortical foci of high connectivity reflect the intrinsic functional organization of the rat brain, with the connectivity properties and node roles in sub-cortical and prefrontal regions being more dependent on the activation of specific neurotransmitter systems, that is, on the specific dynamics of the functional processes elicited by the different pharmacological challenges.

## Figures and Tables

**Table 1:** Summary of global network parameters for the four phMRI networks and the random networks.

Drug	*z*_ thresh_	Power law slope	*K*	*C*	*γ*	*σ*_SW_
Amphetamine	0.71	−1.031	162.3	0.387	3.989	3.011
Fluoxetine	0.94	−0.888	183.1	0.453	3.893	2.836
Nicotine	0.96	−0.671	193.2	0.449	4.627	2.898
Vehicle	0.93	−0.844	177.8	0.422	4.795	2.672

For symbol definitions, see Section 2.

**Table 2:** Differences in degree *k* by VOI compared to vehicle network.

Brain region	VOI	Amphetamine	Fluoxetine	Nicotine
Prefrontal cortex	Cg	+	+++	+++
	mPFC	+	−−−	ns
Somatosensory and motor cortex	M1	+++	+++	+++
	S1BF	+++	+++	+++
	S1FL	+++	+++	+++
Other cortex	ctxV	−−−	+++	+++
	Ins	+++	ns	ns
	PtA	ns	+	+++
Hippocampus	hcAD	−−−	−−−	ns
	hcS	− −	ns	ns
	hcV	−−−	ns	−
Striatum	CPu	ns	+++	−−−
Thalamus	thalDL	−−−	+++	−−−
	thalMD	−−−	++	ns
	thalVM	ns	ns	ns

“+” signs indicate significantly greater *k* compared to vehicle while “−” signs reflect significantly lower *k*, determined in each case using Bonferroni-corrected Mann-Whitney tests: + or − indicates *P*_*c*_ < 0.05, ++ or −− indicates *P*_*c*_ < 0.01, and +++ or −−− indicates *P*_*c*_ < 0.001 (Abbreviations: Cg: cingulate cortex; mPFC: medial prefrontal cortex (prelimbic and infralimbic regions combined); M1: primary motor cortex; S1BF: barrel field of primary somatosensory cortex; S1FL: forelimb field of primary somatosensory cortex, ctxV: visual cortex; Ins: insular cortex; PtA: parietal cortex; hcAD: anterodorsal hippocampus; hcS: subiculum region of hippocampus; hcV: ventral hippocampus; CPu: caudate putamen; thalDL: dorsolateral thalamus; thalMD: mediodorsal thalamus; thalVM: ventromedial thalamus.)

**Table 3:** Differences in clustering coefficient *c* by VOI compared to vehicle network.

Brain region	VOI	Amphetamine	Fluoxetine	Nicotine
Prefrontal cortex	Cg	ns	+++	ns
	mPFC	ns	+++	++
Somatosensory and motor cortex	M1	+++	+++	+++
	S1BF	+++	+++	+++
	S1FL	+++	+++	+++
Other cortex	ctxV	−−−	+++	+++
	Ins	++	ns	+++
	PtA	−−−	+++	+++
Hippocampus	hcAD	−−−	−	ns
	hcS	−−−	+++	++
	hcV	−−−	+++	ns
Striatum	CPu	−−−	+++	+++
Thalamus	thalDL	−−−	+++	+++
	thalMD	−−−	+++	ns
	thalVM	−−−	+++	ns

“+” signs indicate significantly greater *k* compared to vehicle while “−” signs reflect significantly lower *k*, determined in each case using Bonferroni-corrected Mann-Whitney tests: + or – indicates *P*_*c*_ < 0.05, ++ or −− indicates *P*_*c*_ < 0.01, and +++ or −−− indicates *P*_*c*_ < 0.001. (See Table 2 for abbreviation definitions.)
